# The Eyesight

**Published:** 1914-04

**Authors:** Henry Copley Greene


### The Eyesight.

YOUR EYES ARE YOUR BREADWINNERS, TAKE CARE OF THEM.

HENRY COPLEY GREENE.

Take care of your eyes. Among every thousand Americans, one is blind, and many more have too little sight for skilled labor. You yourself are in danger; not serious, perhaps, but real. Your eyesight is not guaranteed. Look ahead. Your eyes are your breadwinners. Take care of them.

WATCH FOR DANGER SIGNALS.

Mothers and fathers, remember that birth is a time of danger. Your child's eyes may easily be infected then or shortly after; and "Babies' Sore Eyes" will result. This redness and swelling may not mean a serious disease. But it is never safe to call babies' sore eyes "just a cold." Your baby may have so severe an eye disease that only prompt, expert care, and experienced nursing can save his sight. Look out for redness and swelling; and call the doctor at once. Better still, ask him to use the harmless preventive (he knows about it) as soon as the baby is born.

HOW ABOUT SCHOOL?

The commonest of serious eye diseases usually attack little children between two and ten years old. So even before they are old enough to go to school, you may find their eyes red and watery. That, too, is a danger signal. Look out for it.

Parents and teachers, when children with red and watery eyes are afraid of the light, be sure that they get good food, and plenty of fresh air; and that they go to a physician who knows about eyes. Otherwise little raised dots may soon appear on the transparent little "portholes" of the eye; ulcers may occur again and again till the little "porthole" looks like ground glass.

EYE-STRAIN.

Even when desks are well lighted, and books are printed in bold type on dull surfaced paper—even then teachers and parents should watch out for eye-strain.

Many children's eyes are not built for school work; they are imperfectly formed and can be made fit for school work only by special glasses. They are short-sighted, or more often far-sighted, or again they are what is called astigmatic. Now without specially prescribed glasses, the far-sighted child and the child with astigmatic eyes may have headaches or fits of dizziness or nausea; and may even lose his health. And without specially prescribed glasses, the near-sighted child may grow more and more near-sighted till everything further off than the end of his nose blurs into a fog.

Look out for signs of eye-strain; the child's face buried in his book, or his forehead puckered in a frown. These "tricks" are generally more than mere habits. They show that the child should be taken to an oculist, who is a physician, for a thorough examination.

GO TO EXPERT PHYSICIANS.

No optician should be trusted to do this work, for in young people, the eye muscles are so taut that no real examination is possible unless they are relaxed with "drops" which only physicians are allowed to use; and only a physician can surely recognize the eye diseases which a child may have. If glasses are ordered, they should be brought back to the physician to see that his prescription has been properly filled. Only in this way can you know that the child has glasses which may give him good health instead of bad, and clear sight instead of defective.

YOUR OWN RISKS.

Too much blindness is needless—the result of carelessness, bad treatment or both. Never neglect an eye that "bothers you." Neglect may result in loss of sight; and when one eye is useless, the other is far more likely to be lost.

ACCIDENTS.

Never let a fellow workman take a splinter out of your eye. His tool or toothpick or handkerchief or tongue may infect your eye. An ulcer may follow, and that often means a totally blind eye. Little things lead to bigger. Delay may spell blindness. For even slight eye accidents go at once to a physician who is an expert in care of the eyes.

DANGEROUS DISEASES.

Many general diseases have bad effects on the eye, which your doctor will do his best to prevent. On the other hand, headaches, dizziness or nausea may point either to the need of eyeglasses, or to the beginnings of serious disease.

If you suffer from these symptoms, or if your sight is failing, go at once to an oculist, who is also a physician. If you need glasses he can prescribe them; if your eyes are diseased, he can advise you. And remember this: that your eye trouble may be the first sign of a dangerous or even fatal disease. Prompt, expert, medical care may save your eyesight and even your life. Be content with nothing less. Take no risks. Don't play with fire. —Selected.

